# Circulating cell free DNA response to exhaustive exercise in average trained men with type I diabetes mellitus

**DOI:** 10.1038/s41598-021-84201-0

**Published:** 2021-02-25

**Authors:** Konrad Walczak, Robert Stawski, Ewelina Perdas, Olga Brzezinska, Piotr Kosielski, Szymon Galczynski, Tomasz Budlewski, Gianluca Padula, Dariusz Nowak

**Affiliations:** 1 Department of Internal Medicine and Nephrodiabetology, Medical University of Lodz, Lodz, Poland; 2 Department of Clinical Physiology, Medical University of Lodz, Lodz, Poland; 3 Department of Rheumatology, Medical University of Lodz, Lodz, Poland; 4 Academic Laboratory of Movement and Human Physical Performance, Medical University of Lodz, Lodz, Poland

**Keywords:** Endocrine system and metabolic diseases, Biochemistry, Molecular biology, Physiology, Biomarkers

## Abstract

It is believed that neutrophils extracellular traps (NETs) formation is responsible for the increase in cf DNA after exercise. Since T1DM is accompanied by enhanced NETs generation, we compared exercise-induced increase in cf DNA in 14 men with T1DM and 11 healthy controls and analyzed its association with exercise load. Subjects performed a treadmill run to exhaustion at speed corresponding to 70% of their personal VO2max. Blood was collected before and just after exercise for determination of plasma cf nuclear and mitochondrial DNA (cf n-DNA, cf mt-DNA) by real-time PCR, blood cell count and metabolic markers. Exercise resulted in the increase in median cf n-DNA from 3.9 ng/mL to 21.0 ng/mL in T1DM group and from 3.3 ng/mL to 28.9 ng/mL in controls. Median exercise-induced increment (∆) in cf n-DNA did not differ significantly in both groups (17.8 ng/mL vs. 22.1 ng/mL, p = 0.23), but this variable correlated with run distance (r = 0.66), Δ neutrophils (r = 0.86), Δ creatinine (r = 0.65) and Δ creatine kinase (r = 0.77) only in controls. Pre- and post-exercise cf mt-DNA were not significantly different within and between groups. These suggest low usefulness of Δ cf n-DNA as a marker of exercise intensity in T1DM men.

## Introduction

Type 1 diabetes mellitus (T1DM) is a serious lifelong disease caused by specific immune-mediated destruction of pancreatic beta cells resulting in a lack of insulin and hyperglycemia^1^. With advancements in disease management, the number of patients with T1DM who are actively and successfully participating in competitive sports is increasing^2,3^. They take part in various sport disciplines including those related to long term strenuous exercise such as triathlon, cycling, running, swimming, basketball or even American football. Moreover, some of them belong to elite athletes who win Olympic or World Cup medals.

Athletes with T1DM may experience almost the same cardio-metabolic health benefits from physical activity as their healthy peers^4,5^. However, strenuous exercise related to training plan or participation in sport competitions may cause an increase in the concentration of circulating pro-inflammatory cytokines^6,7^ and markers of muscle damage^8^, which are the risk factors for the overreaching and decreased performance^9,10^. Recently, it was shown that exercise resulted in the surge of plasma concentration of cell free DNA (cf DNA), independently of workout associated with various sport disciplines (e.g. weightlifting, running, soccer, cycling). Furthermore, the increment of cf n-DNA was always many times higher than the corresponding increments of the standard markers such as lactate or creatine kinase^11–14^.

Circulating cf DNA consists of two pools: cell free nuclear DNA (cf n-DNA) and cell free mitochondrial DNA (cf mt-DNA), deriving from the nucleus or cytoplasmic mitochondria, respectively. Although both cf n-DNA and cf mt-DNA can increase in response to vigorous physical activity, the first one seems to be more affected by exhaustive exercise, including its integrity^8,15^.

Since cf n-DNA rise was observed already after several minutes from the onset of the incremental treadmill run test^16^, it is believed that the source of cf n-DNA is exercise—induced formation of neutrophil extracellular traps (NETs)^17,18^. NETs formation involves the disintegration of nuclear and granular membranes, diffusion of decondensed chromatin into the cytoplasm and its mixing with various proteins. After rupture of neutrophil plasma membrane, this chromatin, associated with various granular proteins, is released into the extracellular space^19,20^ resulting in the increase in circulating cf n-DNA. This process, called NETosis, has various functions including trapping and killing of microbes as well as modulation of immune responses^19^. High glucose concentration in vitro and hyperglycemia in vivo increased NETs formation^21,22^. Moreover, neutrophils isolated from T1DM and type 2 diabetic (T2DM) patients were primed to produce NETs after stimulation with ionomycin (a calcium ionophore) ex vivo and had more peptidyl-arginine-deiminase, an enzyme important in chromatin decondensation and DNA release^23^. Consequently, increased NETosis was reported in T2DM subjects^24,25^, and those with coronary heart disease had increased concentration of circulating cf mt-DNA^26^. Circulating neutrophil elastase and proteinase 3 along with myeloperoxidase-DNA complexes, a well-recognized marker of NETosis^27^, were elevated in T1DM patients especially those with disease duration shorter than 1 year^28^. These results suggest the occurrence of systemically elevated NETosis in T1DM patients which is indicated to be involved in the pathogenesis and complications of this disease^29^. However, results from other studies did not confirm the afore-mentioned findings^30^. On the other hand, it is possible that neutrophils of T1DM patients can be primed for NETs formation and cf DNA release in response to exhaustive exercise. In the light of these facts, it can not be excluded that exercise-induced increase in cf DNA would be higher in T1DM subjects than in healthy controls. NETs are composed of DNA fibers and histones and contain numerous granule proteins. Although citrullination of histones is dispensable for NETs formation, it enhances histone-mediated inflammatory signaling^31^. Circulating extracellular histones are recognized as strong inflammatory mediators^32^ which are involved in the pathogenesis of serious illnesses like trauma-induced multiple organ dysfunction, sepsis or autoimmune diseases^33–35^. Recently, we found that strenuous exercise resulted in parallel substantial increase in the concentration of extracellular citrullinated histone H3 and cf n-DNA in plasma of healthy men^36^. Therefore, if exercise-induced increase in cf n-DNA were higher in T1DM subjects, it would also result in higher increase in extracellular citrullinated histones and possibly stronger inflammatory response after exhaustive exercise. Thus, measurement of post-exercise cf n-DNA would also be an indirect biomarker of exercise-induced systemic inflammatory response. The aims of this study were therefore; (A)—comparison of exercise-induced increment in cf n-DNA (Δcf n-DNA) between physically active young men with T1DM and matched healthy controls; (B)—comparison of pre- and post-exhaustive exercise cf n-DNA and cf mt-DNA plasma levels between these two groups, and (C)—an assessment of the associations between the Δ cf n-DNA, exercise load, and selected parameters of metabolic response to exercise in T1DM volunteers and healthy controls. These could help to estimate plasma cf DNA as a marker of exercise load in sportsmen, especially those with T1DM.

## Results

### Characteristics of the studied groups and exhaustive treadmill run

Table 1 shows characteristics of T1DM subjects and healthy controls. Mean age of healthy controls was higher by 5 years (p = 0.018) than that of T1DM subjects. However, for age range (between 25 and 45 yrs) of studied subjects, this seems to have no clinical significance. Healthy controls had higher plasma cholesterol and triglycerides concentrations (Table 1). Other demographic, physiological, blood cell count and blood chemistry parameters (except of glucose concentration) were not signicicantly different between groups (Tables 2 and 3). All studied men (14 T1DM volunteers and 11 healthy controls) successfully completed the session of exhaustive treadmill exercise. No complications (especially episodes of hypoglycemia in T1DM group) leading to premature cessation of treadmill run were noted during the bout. Although the mean run distance to exhaustion and duration of run tended to be shorter in the T1DM group, the post-exercise heart rate and arterial pressure were similar in both groups (Table 2). Even though subjects were allowed to drink mineral water during the run, exhaustive exercise resulted in a decrease in mean body mass by about 0.7 kg to 0.8 kg in both groups (Table 2). As a consequence, hematocrit, hemoglobin levels, and the number of erythrocytes revealed the tendency to increase in response to exercise (Table 3) as a result of dehydration caused by sweating and hyperventilation. Therefore, all results obtained from plasma or serum analysis were corrected for hematocrit shift related to exercise-induced water loss. White blood cell (WBC) count and the number of granulocytes, lymphocytes, and monocytes raised in a similar extent after the exhaustive bout in both groups; however, the mean exercise-induced increment (Δ) in platelets number was 1.5-times higher (p < 0.05) in T1DM group than in controls (Table 3).

**Table 1:** Characteristic of the studied groups of T1DM volunteers and healthy controls.

Demographic/clinical variables	Volunteers with T1DM	Healthy controls	P
Number of subjects	14	11	–
Age [years]	29.3 ± 5.3	34.0 ± 5.2	0.018
Body mass [kg]	91.0 ± 12.7	87.4 ± 13.8	0.501
Body mass index [kg/m^2^]	28.6 ± 4.0	26.2 ± 3.1	0.317
VO2max [mL/kg*min]	46.7 ± 5.6	49.6 ± 4.5	0.373
Exercise load^†^ [hours/week]	4.6 ± 2.5	5.6 ± 1.6	0.322
Duration of diabetes [years]	15.7 ± 6.1	Not applicable	–
Daily insulin dose [units]	58.1 ± 24.6	Not applicable	–
**Diabetes complications**			
H/R/Nep/Neu	6/4/1/1	Not applicable	–
**Concomitant diseases**			
Utricaria/hypothyroidism	1/2	Not applicable	–
HbA1c [%]	6.7 ± 0.7	Not determined	–
FVC [L]^‡^	5.85 ± 0.70	6.09 ± 0.41	0.373
FEV1 [L]^‡^	4.56 ± 0.65	4.93 ± 0.45	0.066
FEV1/FVC [%]^‡^	78.1 ± 8.1	80.9 ± 5.6	0.149
EF [%]	62.7 ± 5.2	Not determined	–
CRP [mg/L]	1.64 ± 1.35	0.99 ± 0.60	0.267
TC [mmol/L]	4.81 ± 0.82	5.64 ± 0.85	0.029
HDL-C [mmol/L]	1.47 ± 0.30	1.34 ± 0.17	0.202
LDL-C [mmol/L]	2.69 ± 0.67	3.42 ± 0.84	0.044
TG [mmol/L]	1.36 ± 0.56	1.99 ± 0.90	0.050

*T1DM* type 1 diabetes mellitus, *H* arterial hypertension, *R* retinopathy, *Nep* nephropathy, *Neu* neuropathy, *HbA1c* glycated haemoglobin, *FVC* forced vital capacity, *FEV1* forced expiratory volume in the first second, *EF* ejection fraction, *CRP-C* reactive protein, *TC* total cholesterol, *HDL-C* high-density lipoprotein cholesterol, *LDL-C* low-density lipoprotein cholesterol, *TG* triglycerides.

^†^Cross country running, treadmill running or soccer.

^‡^Similarly, no differences were noted when the parameter was expressed as percent of predicted.

**Table 2:** Parameters monitored during bouts of exhaustive treadmill run.

Parameter	Bout of exhaustive treadmill exercise		
	T1DM male volunteers	Healthy male controls	P
Run distance to exhaustion [km]	6.5 ± 4.5	8.6 ± 5.5	0.244
Run time [min]	39 ± 29	47 ± 31	0.647
Baseline heart rate [beats/min]	77 ± 9	72 ± 11	0.120
heart rate at the end of run [beats/min]	185 ± 12	184 ± 10	0.727
% of maximal heart rate at the end of run *	96.9 ± 6.6	99.4 ± 5.9	0.33
Baseline blood pressure [mmHg] S/D	129 ± 9/85 ± 7	127 ± 6/80 ± 4	0.344/0.066
Blood pressure after exercise [mmHg] S/D	157 ± 17/83 ± 7	172 ± 20/82 ± 11	0.107/0.893
Loss of body mass [kg]	0.84 ± 0.66	0.73 ± 0.65	0.609

*S* systolic, *D* diastolic, After determination of VO2max at day 1, treadmill exercise test to exhaustion at speed corresponding to 70% of personal VO2max was performed at day 7. Results are expressed as mean ± SD.

*Calculated according to Fox formula.

**Table 3:** Changes of blood cell count, selected circulating markers of muscle damage and metabolic response to exercise in average-trained males with TIDM and healthy controls after exhaustive treadmill run.

Variable	Bout of exhaustive treadmill exercise					
	T1DM male volunteers			Healthy male controls		
	Before	After	Increment (Δ)	Before	After	Increment (Δ)
Hct [%]	45.6 ± 2.3 (45.3)	46.7 ± 2.8 (45.9)	1.1 ± 1.9 (1.0)	46.5 ± 3.4 (48.6)	48.1 ± 3.4 (48.1)	1.4 ± 1.3 (1.5)
Hgb [g/dL]	15.4 ± 0.8 (15.2)	15.8 ± 1.0* (15.9)	0.4 ± 0.6 (0.4)	15.6 ± 1.1 (16.0)	16.1 ± 1.2 (15.9)	0.4 ± 0.4 (0.4)
RBC [× 10^6^/mm^3^]	5.06 ± 0.23 (5.08)	5.19 ± 0.32 (5.16)	0.12 ± 0.2 (0.08)	5.17 ± 0.4 (5.08)	5.32 ± 0.42 (5.23)	0.15 ± 0.13 (0.13)
WBC [× 10^3^/mm^3^]	6.50 ± 1.33 (6.20)	9.29 ± 2.43* (9.60)	2.79 ± 1.43 (2.70)	5.86 ± 0.6 (5.61)	9.45 ± 1.94* (9.32)	3.59 ± 1.57 (3.60)
GRA [× 10^3^/mm^3^]	4.56 ± 1.25 (4.30)	5.83 ± 1.64* (6.00)	1.27 ± 0.58 (1.30)	3.98 ± 0.7 (3.91)	6.02 ± 1.88 (5.43)	2.04 ± 1.43 (1.50)
MON [× 10^3^/mm^3^]	0.22 ± 0.11 (0.20)	0.35 ± 0.17* (0.30)	0.13 ± 0.12 (0.10)	0.20 ± 0.0 (0.21)	0.36 ± 0.08 (0.42)	0.16 ± 0.09 (0.20)
LYM [× 10^3^/mm^3^]	1.72 ± 0.44 (1.70)	3.12 ± 1.35* (2.90)	1.39 ± 0.98 (1.10)	1.68 ± 0.3 (1.63)	3.07 ± 0.81 (2.91)	1.39 ± 0.70 (1.30)
PLT [× 10^3^/mm^3^]	236 ± 51 (212)	313 ± 62* (308)	77 ± 35 (77)	208 ± 33 (199)	258 ± 40* (248)	50 ± 21^†^ (49)
CK [U/L]	267 ± 175 (199)	325 ± 211* (235)	58 ± 48 (41)	162 ± 63 (141)	211 ± 112* (179)	48 ± 81 (32)
AST [U/L]	31 ± 13 (31)	34 ± 15 (31)	3 ± 4 (3)	26 ± 7 (25)	35 ± 19 (30)	9 ± 14 (5)
ALT [U/L]	27 ± 12 (25)	29 ± 12 (26)	2 ± 3 (1)	28 ± 8 (26)	33 ± 14 (28)	6 ± 9 (3)
Lactate [mmol/L]	2.0 ± 0.7 (1.9)	8.3 ± 3.5* (7.8)	6.3 ± 3.6 (5.6)	1.7 ± 0.8 (2.1)	8.9 ± 4.6* (8.0)	7.2 ± 4.9 (5.9)
Creatinine [µmol/L]	89.9 ± 10.2 (90.0)	107.6 ± 13.2* (110.0)	17.6 ± 8.4 (14.5)	84.8 ± 11(81.0)	116.0 ± 6.8* (112.1)	31.2 ± 12.1 (32.0)
Urea [mmol/L]	5.9 ± 1.2 (5.6)	6.3 ± 1.1* (6.0)	0.4 ± 0.2 (0.3)	5.8 ± 1.2 (5.6)	6.4 ± 1.5* (5.8)	0.6 ± 0.4 (0.5)
Glucose [mmol/L]	11.7 ± 3.8 (10.8)	9.7 ± 3.9 (10.1)	-2.0 ± 4.6 (-1.1)	5.2 ± 0.8^†^ (5.6)	6.8 ± 1.8*^†^ (6.1)	1.6 ± 2.0^†^ (1.1)
CRP [mg/L]	1.64 ± 1.35 (1.25)	1.69 ± 1.34 (1.47)	0.05 ± 0.17 (0.05)	0.99 ± 0.6 (0.80)	1.53 ± 1.28* (1.17)	0.54 ± 0.89^†^ (0.20)

*T1DM* type 1 diabetes mellitus, *Hct* hematocrit, *Hgb* hemoglobin, *RBC* red blood cells, *WBC* white blood cells, *GRA* granulocytes, *MON* monocytes, *LYM* lymphocytes, *PLT* platelets, *CK* creatine kinase, *AST* asparate aminotransferase, *ALT* alanine aminotransferase, *CRP-C* reactive protein. Results are expressed as mean ± standard deviation and (median in parentheses). Other details as for Table 2.

*– vs corresponding value before the bout, p < 0.05.

^†^– vs corresponding value in T1DM group, p < 0.05.

### Markers of muscle damage and metabolic response to exhaustive treadmill run

Significant increase in circulating CK (but not in AST and ALT) was observed after exercise in T1DM volunteers. The concentrations of lactic acid, creatinine and urea increased significantly by 4.1-, 1.2- and 1.1-times in response to exercise in this group (Table 3).

These variables behaved quite similarly in healthy controls. Mean post-exercise glucose levels tended to decrease in T1DM subjects while in healthy controls exercise caused significant increase in glucose concentration by about 1.3-times (Table 3). Consequently, Δ glucose differed significantly between T1DM subjects and healthy controls (− 2.0 ± 4.6 mmol/L vs. + 1.6 ± 2.0 mmol/L). In contrast to T1DM, mean CRP concentration increased in response to exercise in healthy controls (Table 3).

### Changes of plasma concentrations of cell free nuclear and mitochondrial DNA in response to exhaustive treadmill run

**Figure 1:**
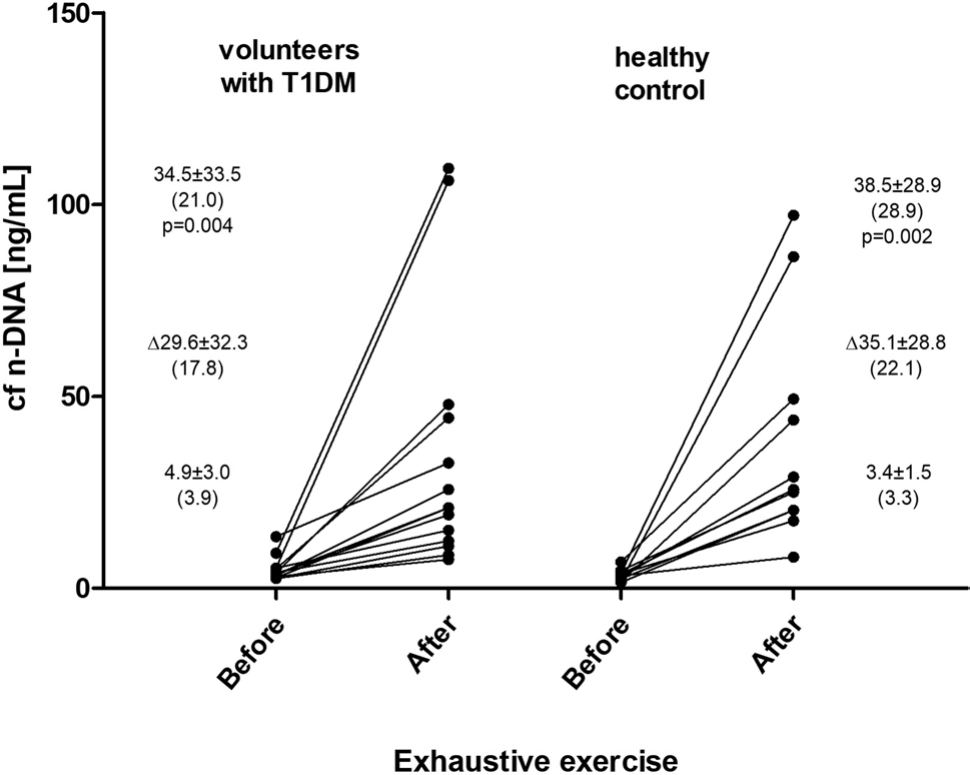
Circulating cell free nuclear DNA (cf n-DNA) response to exhaustive treadmill exercise at speed corresponding to 70% of personal VO2max in male volunteers with T1DM (n = 14) and healthy control group (n = 11). *Δ* exercise induced increment. Results are expressed as mean, standard deviation, and (median). Exercise resulted in significant increase in cf n-DNA in both groups. No significant differences were noted between groups. The points (corresponding to values before and after the exercise) joined with lines represent individual results in both studied groups.

**Figure 2:**
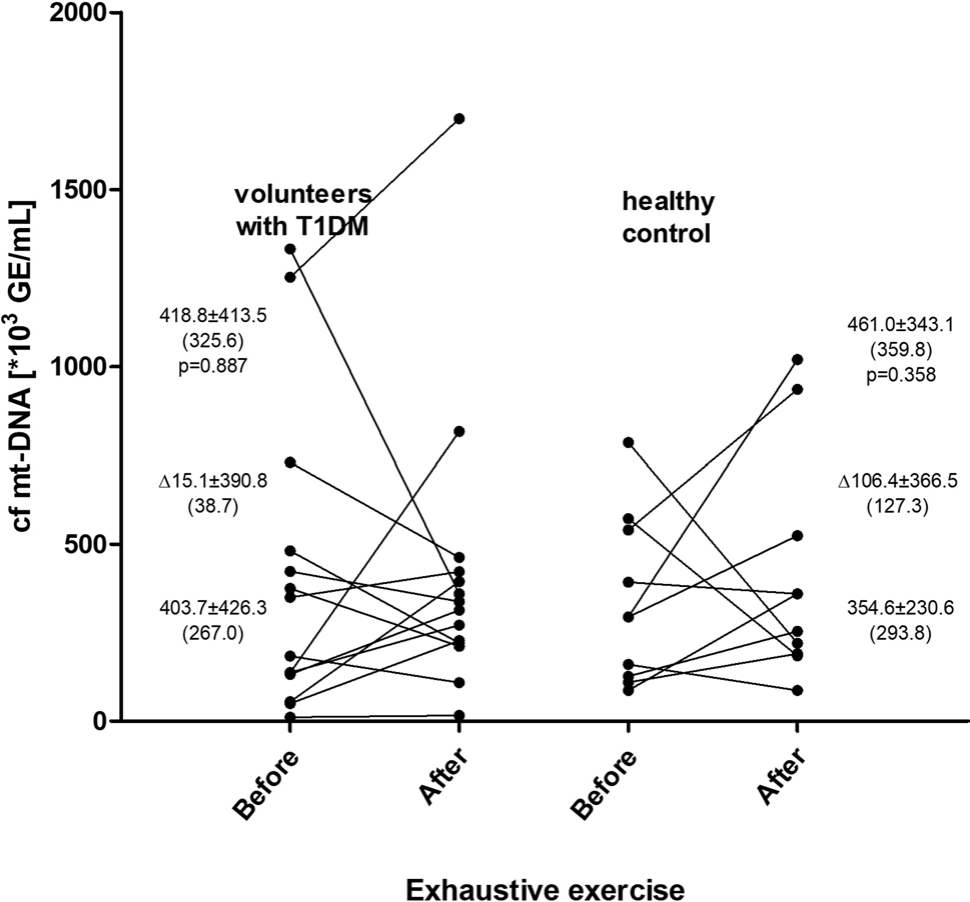
Circulating cell free mitochondrial DNA (cf mt-DNA) before and after exhaustive treadmill exercise at speed corresponding to 70% of personal VO2max in male volunteers with T1DM (n = 14) and healthy control group (n = 11). *Δ* exercise induced increment. Results are expressed as mean, standard deviation, and (median). No significant differences were found within and between groups. The points (corresponding to values before and after the exercise) joined with lines represent individual results in both studied groups.

Mean pre-exercise plasma concentration of cf n-DNA was 4.9 ± 3.0 ng/mL in T1DM subjects. Exhaustive treadmill run resulted in great elevation of mean concentration of circulating cf n-DNA (by 7.1-times) which reached 34.5 ± 33.5 ng/mL (Fig. 1). Mean pre- and post-exercise plasma levels of cf n-DNA were similar in T1DM subjects and in healthy controls. Thus, exercise-induced increment (Δ) in circulating cf n-DNA was not significantly different between the studied groups (29.6 ± 32.3; Me 17.8 ng/mL for T1DM subjects vs. 35.1 ± 28.8; Me 22.1 ng/mL for healthy controls, p = 0.23) showing almost the same intensity of cf n-DNA response to exercise in T1DM subjects and healthy controls (Supplemental Table S1). Baseline, and pre-exercise levels of cf mt-DNA were not significantly different between the studied groups (Fig. 2). In contrast to cf n-DNA, cf mt-DNA did not increase after exercise either in T1DM subjects or healthy controls (Fig. 2).

### Correlations of exercise-induced increment in cf n-DNA (Δcf n-DNA) with selected markers of muscle damage and metabolic response to exercise

Δcf n-DNA positively correlated with run distance to exhaustion, pre- and post-exercise WBC and neutrophils as well as with exercise-induced increment (Δ) in WBC, neutrophils, CK and creatinine in healthy controls (Table 4, Supplemental Figs. S1–S10, Supplemental Table S5). However, in the group of T1DM volunteers no significant correlations between Δcf n-DNA and these variables were noted. There was no significant association between Δcf n-DNA and VO2max as well as between Δcf n-DNA and Δ lactate in both groups (Table 4). Neither pre-exercise cf mt-DNA nor post exercise cf mt-DNA associated with any measured variables in healthy controls and T1DM subjects.

**Table 4:** Correlations (r) between exercise-induced increment in cf n-DNA (Δ cf n-DNA) and selected variables in the group of T1DM volunteers (n = 14) and healthy controls (n = 11).

Variable	Exercise induced increment in cf n-DNA	
	T1DM volunteers (n = 14)	Healthy controls (n = 11)
Run distance to exhaustion	0.09 P = 0.760	0.66 P = 0.026
Pre-exercise glucose	− 0.31 P = 0.283	0.01 P = 0.974
Pre-exercise WBC	0.19 P = 0.529	0.64 P = 0.032
Pre-exercise neutrophils	0.29 P = 0.338	0.63 P = 0.040
Post-exercise WBC	0.02 P = 0.944	0.75 P = 0.007
Post-exercise neutrophils	0.23 P = 0.438	0.88 P = 0.0003
Δ WBC	− 0.14 P = 0.642	0.67 P = 0.023
Δ neutrophils	0.05 P = 0.883	0.86 P = 0.001
Δ CK	0.10 P = 0.746	0.77 P = 0.005
Pre-exercise lactate	− 0.28 P = 0.323	− 0.17 P = 0.608
Post-exercise lactate	− 0.37 P = 0.189	− 0.53 P = 0.090
Δ lactate	− 0.30 P = 0.294	− 0.50 P = 0.115
Pre-exercise creatinine	0.22 P = 0.457	− 0.71 P = 0.015
Post-exercise creatinine	0.23 P = 0.435	− 0.11 P = 0.755
Δ creatinine	0.09 P = 0.747	0.65 P = 0.030
VO2max	0.23 P = 0.42	0.27 P = 0.94

*WBC* white blood cell, *CK* creatine kinase, *Δ* exercise induced increment in given variable, other details as for Table 2. Due to technical problems, one subject with T1DM had no blood cell count. Therefore, correlations between Δ cf n-DNA and WBC as well between Δ cf n-DNA and neutrophils were calculated from 13 cases for the T1DM group.

## Discussion

Nowadays it is suggested that regular physical activity could be beneficial to health of subjects with T1DM^37^. This practice can decrease the risk factors for cardiovascular diseases, lowers blood glucose levels, reduces body fat, increases mass of skeletal muscles, and improves cardiovascular function and physical efficiency. However, when exogenic levels of insulin are to high, hypoglycemia may occur during or after exercise. And, conversely, when there is a lack of insulin, exercise can lead to hyperglycemia or ketosis^37^. Therefore, it is worthy and interesting to study various biochemical and molecular (including cf DNA) parameters of body response to exercise to optimize exercise intensity in T1DM subjects. We found that a single bout of exhaustive treadmill run resulted in a distinct increase in cf n-DNA in average trained men with T1DM. Although T1DM subjects tended to run a shorter distance than healthy controls, their cf n-DNA response to exercise was comparable to that observed in this latter group. Moreover, cf mt-DNA was not altered by exercise in both healthy men and T1DM volunteers. These results indicate that cf DNA response to exercise (composed of cf n-DNA and cf mt-DNA changes) is similar in T1DM subjects and healthy controls. Exhaustive treadmill run increased cf n-DNA by 5.4- and 8.7-times in the T1DM group and healthy controls, respectively. These relative increases in cf n-DNA are similar to that caused by 10 km cross country run (7.6-times) and by short-term treadmill exercise (9.9-times) with velocity increased by 2 km/h every 3 min until subject exhaustion^18^. In another study, incremental treadmill test until volitional exhaustion caused 9.8-fold increase in cf DNA in the group of handball players and triathletes^16^. However, just after half- marathon (about 21.09 km) circulating cf DNA increased much more, about 18-times^38^.

Strong correlations between Δ cf n-DNA and Δ neutrophils, as well as between Δ cf n-DNA and pre-and post-exercise neutrophils in healthy controls are in line with the opinion that NETosis is a source of elevated post-exercise cf n-DNA^16–18^. Surprisingly, we did not observe such associations in T1DM subjects. Taking all together, these results suggest that circulating neutrophils of T1DM subjects were not primed for an increased release of n-DNA in response to exhaustive run, and perhaps the process of release of cf n-DNA is not exactly the same as in healthy controls. Activation of various molecular pathways (e.g. NADPH oxidase-dependent and NADPH oxidase-independent) may induce NETs formation and chromatin externalization resulting in an increase in circulating cf DNA^19,20^. Regardless of the initiating signal, the involvement of nuclear, granular and plasma membranes (their rupture and reorganization) is a common feature of NETosis^19^. Plasma membranes of blood cells isolated from DM patients were found to be stiffer and less fluid due to protein glycation and oxidative stress^39–41^. Moreover, neutrophils of T1DM subjects had altered numerous functions including adhesion, migration, oxidative burst and microbicidal activities^42^. These observations may partly explain no associations between Δ cf n-DNA and pre-, post-exercise neutrophils and Δ neutrophils in T1DM subjects, although the intensity of cf DNA response to exercise was similar in the T1DM group and healthy controls.

Other mechanisms such as the release of exosomes or microvesicles and the active release of DNA can contribute to rapid increase in cf DNA during exercise^43^. Moreover, binding of released n-DNA to outer membranes of blood cells can affect the intensity of cf DNA response to exercise^43^. It can not be excluded that T1DM can alter these processes and be an additional cause of the different results of the afore-mentioned correlation analyses in T1DM subjects and healthy controls. Concomitant treatment with Ramipril in 6 of 14 subjects with T1DM could be another factor responsible for the low degree of association between Δcf n-DNA and variables in reaction to exhaustive run. In vitro, Ramipril enhanced by about 1.3-times the release of myeloperoxidase from isolated resting and zymosan-stimulated human neutrophils, However, this did not exceed 6% and 10% of the total enzyme content, respectively^44^. On the other hand, treatment with Ramiprill suppressed the increase in plasma concentration of interleukin 6 and TNFα related to coronary artery bypass grafting in patients with coronary heart disease^45^. Both these cytokines can induce NETs formation with subsequent cf DNA release^21,46^. Thus, Ramipril can evoke opposing processes in respect of activation of human neutrophils. However, this issue requires further studies involving T1DM subjects with and without treatment with Ramipril.

Exercise resulted in significant decrease in glucose levels in T1DM subjects while in healthy controls the opposite tendency was observed. There are numerous mechanisms to maintain euglycemia in healthy subjects. Although exercise stimulates muscle glucose uptake mainly by translocation of active GLUT-4 to the outer cell membranes, other reactions and processes such as inhibition of insulin release, increased secretion of glucagon, catecholamines and cortisol leading to hepatic gluconeogenesis and glycogenolysis in muscles and liver prevent hypoglycemia and even can moderately increase glucose levels^37,47^. In T1DM subjects, hepatic glucose output is too low due to inadequate counterbalanced hormonal response and blood insulin (exogenous) could be too high causing the decrease in post-exercise glucose levels^37^. However, it should be pointed out that all T1DM subjects modified insulin dose before the bout of exercise and, therefore, none of them experienced symptoms of hypoglycemia.

Both groups had similar pre-exercise concentration of plasma CRP and WBC including neutrophils. The exercise-induced Δ WBC and Δ neutrophils were also comparable in these groups, while CRP increased significantly only in healthy controls. This acute phase protein is primarily synthetized in liver hepatocytes. Liver was described to have lower responsiveness to catecholamines and other hormones in T1DM subjects^37,48^. Perhaps, this may explain the afore-mentioned differences. On the other hand, Ramipril was reported to decrease CRP levels by about 24% and 32% in diabetic and atherosclerotic patients, respectively^49^. Because part of T1DM patients received Ramipril due to arterial hypertension, this treatment might have also contributed to low CRP response to exercise in this group.

We initially assumed that patients with T1DM would have had baseline (pre-exercise) cf n-DNA higher than healthy controls. Instead, baseline cf n-DNA did not differ significantly between these groups. This indicates that, in subjects with T1DM without serious complications and inflammatory processes, there is no enhancement of cf n-DNA release. Consequently, T1DM as such did not elevate cf n-DNA in circulating blood.

Metabolic alterations and impaired innervation of skeletal muscles related to lack of insulin may lead to their decreased contractility in patients with T1DM^37,50^. Moreover, the whole body fatigue is a frequent compliant in diabetic patients^51^, and scores of perceived exertion during continuous prolonged exercise are higher in T1DM subjects than in healthy controls^52^. In addition, T1DM subjects fatigued significantly sooner (by about 45%) than healthy controls as evaluated during isometric exercise fatigue session with the surface electromyography and intramuscular signals recording^50^. These findings may explain to some extent the shorter run distance to exhaustion (at the same relative intensity) and run time in the T1DM group than in healthy controls. Although these differences were not statistically significant, they may also contribute to the negative results of correlation analysis between Δ cf n-DNA and exercise load in the T1DM group. It should be pointed out that the run distance to exhaustion and run time had strong variability in both groups. Run distance to exhaustion coefficient of variation was 69.2% and 63.9%, while run time coefficient of variation was 74.3% and 65.9% in T1DM subjects and healthy controls, respectively. This difference indicates that individual volunteers ran different distances at the same relative intensity of exercise and their energy expenditure during the test was also different. These outcomes are in agreement with the previous study showing a large range of time to exhaustion (from 3 to 36 min) under conditions of cycloergometer test with constant load corresponding to 80% VO2max in a group of male trained cyclists^53^. Other authors also reported a great inter-individual variability of endurance within the group of competitive cyclists with an comparable VO_2_max^54^. Additionally, the coefficient of variation of post-exercise lactate was 42.2% and 51.7% in T1DM men and healthy controls. Comparable heterogeneity of lactate response was observed after 60 min of cycloergometer test with constant load corresponding to 60% or 75% VO2max in healthy men^55^. Perhaps, a better approach in the studies on cf DNA response to exercise would be an application of treadmill run resulting in the same energy expenditure in all subjects rather than a treadmill run to exhaustion. Possibly, the best way to solve this issue is the execution of both these tests separated by sufficient resting time.

Acute exhaustive exercise (rowing ergometer, mean duration 21 min) caused a transient increase in cf DNA which returned to the baseline level at 30 min-post exercise in highly trained male rowers^56^. This rapid cf DNA clearance may result from exercise-induced release of DNAse^56^, yet renal and liver elimination could also play some role^57^.

In order to partially limit the effect of cf-DNA clearance on the magnitude of cf n-DNA response to exercise, we applied a treadmill exhaustive run at relative high intensity corresponding to 70% of VO2max. Thus, the mean run duration did not exceed 40 min and 50 min in T1DM subjects and healthy controls, respectively. This exercise intensity was close to the lactate threshold of examined participants (Supplemental Tables S4 and S5), and exercise bout resulted in about 4-times increase in median plasma lactate. Such rise in lactate was observed during 40 min of continuous exercise with constant comparable load^58^.

Glucose at concentrations ranging from 15 mmol/L to 30 mmol/L induced in vitro the formation of NETs and, in consequence, the release of cf n-DNA^21,22,25,59,60^ (Supplemental Table S4). Median pre- and post-exercise glucose concentration in T1DM subjects were 10.8 mmol/L and 10.1 mmol/l, and although abnormally elevated, they were far below the afore-mentioned values which stimulated NETs formation in experimental studies. These results may explain the lack of correlations between Δ cf n-DNA and glucose concentrations in the studied subjects, and may also contribute to similar pre-exercise cf n-DNA levels in both groups.

Another factor related to T1DM which can stimulate NETs formation is homocysteine. In vitro, incubation of neutrophils from healthy subjects with homocysteine at concentrations from 50 to 500 µmol/L caused a formation of NETs which reached plateau at concentration ≥ 100 µmol/L^60^. Of T1DM patients only those with retinopathy or nephropathy had elevated homocysteine levels in comparison to healthy controls^61^. Four of our studied T1DM patients had these complications (Table 1). However, because we did not measure plasma homocysteine, its contribution to pre-exercise cf n-DNA concentrations and Δ cf n-DNA in the T1DM group can not be clearly judged. Exhaustive exercise did not increase cf mt-DNA in T1DM subjects and healthy controls. In a previous study, there was a significant decrease in circulating cf mt-DNA in healthy moderately trained men who completed 90 min treadmill run at speed corresponding to 60% of their VO2max, which normalized after 40 min of recovery^62^. Recently, no significant alterations of cf mt-DNA levels were described in blood samples taken from healthy volunteers when they reached exhaustion during controlled ergospirometry cycle test^63^. However, cf mt-DNA rose significantly in post-exercise samples taken after 30 and 90 min of rest^63^. These evidences indicate that the time of blood collection has great importance on the results of studies on exercise-induced cf mt-DNA changes. Nevertheless, none of the afore-mentioned studies revealed significant increase in cf mt-DNA in blood samples collected just after exercise.

T1DM patients, especially those with disease duration shorter than 1 year, had elevated circulatory markers of NETosis such us neutrophil elastase and proteinase 3. These markers were markedly decreased in patients with disease duration longer than 5 years (median 7.6 years) but were still higher than in healthy controls^28^. We studied a group of T1DM men with a mean disease duration of about 16 years. They had pre-exercise cf n-DNA levels and Δ cf n-DNA similar to healthy controls. These outcomes suggest the involvement and activation of neutrophils, including NETs formation at the onset of T1DM, while, with several years of disease duration, the intensity of baseline and probably exercise-induced NETosis and release cf n-DNA returned to normal.

It is proposed that exercise-induced increase in cf DNA could be a marker of a single training load session^13,64^. This assertion is based on the results of previous studies showing a positive association of post-exercise cf DNA with the duration and the intensity of aerobic running^65^, and with markers of muscle damage^64^. Moreover, elevated post-exercise cf DNA was shown to have predictive potential of muscle-performance decrease within 2 days after a bout of heavy resistance exercise^66^. Significant correlations of Δ cf n-DNA with run distance, Δ CK and Δ creatinine in healthy subjects are in line with these observations, and support the concept of cf DNA as a new additional and supportive molecular tool to measure training and competition intensity in sportsmen or physically active subjects without concomitant diseases^13,43^. Although the percentage increment in cf n-DNA was the highest one of the analyzed set of markers of muscle injury and metabolic response to exercise in both studied groups (Supplemental Table S5), the lack of the afore-mentioned relationships in the T1DM group limits the diagnostic and predictive value of cf n-DNA determination in physically active men with T1DM. On the other hand, it should be pointed out that precise evaluation of body response to exercise involves analysis of physiological measures, a panel of metabolic, inflammatory and hormonal markers, and assessments of subjective perception of effort during exercise including the Borg’s rating of perceived exertion. Therefore, exercise-induced Δ cf n-DNA as an additional objective measure of exercise load ought to be interpreted in relation to the other above-noted variables together with the duration, intensity and type of physical activity. A relatively low number of studied subjects, inclusion of only male volunteers, analysis of two time-points (before and just after the exhaustive treadmill run), and no measurement of subjective exercise intensity with a Borg’s rating of perceived exertion scale are the limitations of our study protocol. Initially, the project included male and female recreational runners and collection of blood samples at least twice, during the run and after 30 min recovery. Although inclusion criteria were not restrictive, it was not possible to recruit female runners with T1DM. Additionally, the majority of volunteers, especially those with T1DM, did not accept numerous blood sampling even with the use of intravenous cannula. That is why the study included only male volunteers and examined two-time points which precluded intra- and between-groups analysis of kinetics of cf DNA response to exercise. Moreover, due to ethical reasons, we could not stop ongoing treatment (insulin, Ramipril) in T1DM volunteers. Perhaps, a larger number of studied T1DM subjects would be sufficient to solve the issue of plausible effect of Ramipril on cf n-DNA response to exercise in this group. Analysis of correlations between exercise-induced Δ cf n-DNA and perceived effort estimated by the Borg’s scale would be interesting because, to the best of our knowledge, such results were not published so far. In conclusion, we found that T1DM volunteers had a similar intensity of cf n-DNA response to a single bout of exhaustive exercise as healthy controls. However, opposite to healthy subjects, Δ cf n-DNA in the T1DM group did not correlate with the run distance to exhaustion and markers of metabolic response to exercise. Therefore, the usefulness of cf n-DNA as an additional marker of exercise load in physically active men with T1DM seems to be limited and requires further studies on a large number of T1DM volunteers (especially those free of any medication, except for insulin) subjected to bouts of exercise with various loads and duration to solve this issue.

## Material and methods

### Studied population

The study included 25 average-trained men, 14 with T1DM and 11 apparently healthy controls, all recreational runners and training consistently for more than 6 years (Table 1). Volunteers with T1DM were recruited from the Diabetes Clinic, (Central Clinical Hospital of the Medical University of Lodz, Poland) while healthy controls were from the database of the Academic Laboratory of Movement and Human Physical Performance “DynamoLab” (Medical University of Lodz, Poland). Both groups were matched for the training volume (hours per week) and had to fulfill the following inclusion criteria: age between 25 and 45 years, body mass index between 18.5 kg/m^2^ and 35 kg/m^2^, and a written informed consent before initiating the study procedures. The exclusion criteria included: presence of contraindications to exhaustive exercise, alcohol and illicit drug abuse, current cigarette smoking, any history of acute infectious or inflammatory diseases, use of any vitamins or food supplements, or any systemic pharmacological treatment within 3 months prior to the study. For subjects with T1DM, presence of complications of diabetes (not related to systemic chronic inflammatory processes) and their pharmacological treatment as well as arterial hypertension controlled with Ramipril (a second generation angiotensin converting enzyme inhibitor) in a daily dose not exceeding 10 mg were allowed. All subjects agreed to keep their dietary habits constant during the study period and to comply with the instructions related to participation in the study.

### Study protocol

The study consisted of 2 visits at the 1st and 7th day of observation. All visits started at 9:00 AM and included medical examination, arterial blood pressure measurement, and resting electrocardiography to exclude any contraindications to exercise test. At the first visit (day 1st), subjects underwent spirometry tests, echocardiographic determination of ejection fraction (only in volunteers with T1DM as an additional safety parameter) and treadmill VO2max test. Afterwards, at the second visit (day 7th), participants performed treadmill exercise to volitional exhaustion at speed corresponding to 70% of their personal VO2max. Pre- and immediately post-exhaustive exercise venous blood samples (15 mL) were collected into vacutainer tubes (Becton Dickinson, Franklin Lakes, NJ) with EDTA for cf DNA extraction and blood cell count determination, into tubes containing gel and clot activator for blood chemistry, and into tubes with sodium oxalate and potassium fluoride for lactate determination. All exercise bouts were performed at the Academic Laboratory of Movement and Human Physical Performance “DynamoLab” of the Medical University of Lodz (ambient temperature 20–21 °C, relative air humidity 50% to 60%). During the 7 days of study, volunteers did not perform any exhaustive exercise besides those related to the study protocol. The study was conducted according to the Declaration of Helsinki. The protocol was reviewed and approved by The Medical University of Lodz Ethics Committee (RNN/95/14/KB), and all volunteers provided written informed consent. Additionally, a separate written informed consent for VO2max test and exhaustive treadmill exercise was obtained from all studied volunteers as described previously^8^.

### Determination of VO2max

VO2max was determined by a continuous incremental maximal exercise test on a programmable treadmill (Trackmaster CP 425) interfaced with a Ultima CardiO2 PFX (gas exchange analysis system) integrated with a 12-lead wireless Mortara ECG (Medical Graphics Corporation, St Paul, MN, USA), as described previously in details^8^. Only one subject was examined per day, and the gas exchange analysis system was calibrated before each exercise test according to manufacturer instruction. Medgraphics Breeze Suite 7.2.0 software was used for ergospirometric data collection and calculation. Individual results of VO2max test are shown in Supplemental Tables S2 and S3.

### Execution of treadmill exhaustive exercise

All subjects had usual breakfast between 7:00 and 8:00 AM. Subjects with T1DM took insulin dose calculated on the basis of their individual carbohydrate intake and participation in the exhaustive exercise (flexible dose therapy). At 10:00 AM (after all physical exams and exclusion of contraindications to exercise testing) subjects started to run on the treadmill until volitional exhaustion. The treadmill constant inclination was 1.5% and the speed was set at the value corresponding to 70% of personal VO2max. This value was determined for each volunteer during the measurement of VO2max at the 1st visit. Volitional exhaustion was defined as subjects inability to maintain the required exercise intensity (run at constant speed of the treadmill) or their wish to stop the test, despite strong encouragement to continue by the testing staff. Heart rate was monitored with the Polar chest strap H7 heart rate sensor (Polar Inc., Kempele, Finland). Subjects were allowed to drink only mineral water during the test. Body weight and blood pressure were measured as well as venous blood samples were taken before and just after the exhaustive treadmill run as described previously^8,15^.

### Cell free DNA extraction

Plasma from EDTA blood samples was obtained by centrifugation (1 600 × g, 4 °C) for 10 min. Then, plasma samples were subjected to a second centrifugation (16,000×*g*, 4 °C, 5 min) to remove the cell debris and stored at − 80 °C for no longer than 4 weeks until cf DNA isolation and measurement of cf n-DNA and cf mt-DNA levels. Cf DNA was isolated from 400 μL plasma samples using a QIAamp DNA Blood Mini Kit (Qiagen GmbH, Hilden, Germany) according to manufacturer instructions with elution into 40 μL TE buffer as decribed in previous studies^8,15^.

### Measurement of cf n-DNA and cf mt-DNA in plasma

For the quantification of isolated from plasma cf n-DNA and cf mt-DNA, a quantitative real time PCR (qPCR) was performed at the Central Scientific Laboratory “CoreLab” of the Medical University of Lodz (Lodz, Poland) using the glyceraldehyde 3-phosphate-dehydrogenase (GAPDH) gene for cf n-DNA, and mitochondrially encoded ATP synthase 8 (MT-ATP 8) gene for the cf mt-DNA. The sequences of the primers and execution of calibration curve were described in our previous studies^8,15,67^. Simultaneous multiplex qPCR was carried out in 20 μL of total reaction volume containing 5 μL H2O, 10 μL TaqMan1Universal PCR Master Mix (Applied Biosystems, Branchburg, New Jersey, USA), 0.25 μL of each of the four afore-mentioned primers (Sigma-Aldrich, St. Louis, Missouri, USA), 1 μL of a FAM-labeled MT-ATP 8-probe, 1 μL of a MVIC-labeled GAPDH probe (both probes from Applied Biosystems, Branchburg, New Jersey, USA), and 2 μL of TE buffer containing cf DNA isolated from plasma. The final concentrations of primers and probes were 0.6 μmol/L and 0.4 μmol/L, respectively. Negative control samples received 2 μL of TE buffer without cf DNA from plasma. All reactions were performed in duplicate using the 7900 HT Real-time PCR System (Applied Biosystems, Branchburg, New Jersey, USA) under the following conditions: an initiation step at 50 °C for 2 min followed by a first denaturation at 95 °C for 10 min, then 40 cycles of 95 °C for 15 s and annealing at 60 °C for 1 min. Individual results were obtained as a mean from two separate runs and expressed in ng/mL for cf n-DNA and as genome equivalents (GE)/mL plasma (1 GE = 6.6 pg DNA) for cf mt-DNA as described previously^8,15^.

### Other determinations

Complete blood count, lipid panel (total cholesterol—TC, high-density lipoprotein cholesterol—HDL-C, low-density lipoprotein cholesterol—LDL-C, triglicerydes—TG), serum activities of creatine kinase (CK), asparate aminotransferase (AST), alanine aminotransferase (ALT), and concentrations of C-reactive protein (CRP), lactic acid (enzymatic method, Beckman Coulter AU analyzer), glucose, urea, creatinine and glycated haemoglobin (HbA1c, only in subjects with T1DM) were determined at the Diagnostic Laboratory of the Central Clinical Hospital of the Medical University in Lodz (Lodz, Poland) as described previously^8^.

### Statistical analysis

Results are expressed as a mean (SD) and median (Me) and interquartile range (IQR). Analysis of variance (ANOVA) with Scheffe´ Post Hoc test for repeated observations (parametric test) or Friedman's ANOVA with post hoc analysis (non-parametric test) was applied for the assessment of changes in variables over time (before and after exercise bout) depending on data distribution, which was tested with Shapiro–Wilk's W test (STATISTICA 13.1 StatSoft software). Differences between groups (T1DM group vs. healthy controls, single comparison at one time point) were evaluated with the Student t-test and U Mann–Whitney test depending on the normality of distribution and equality of variance. Pearson linear correlation with t-test were used for analysis associations between Δ cf n-DNA and selected parameters of muscle damage, blood cell count and metabolic response to exercise. A p value < 0.05 was considered significant.

## Supplementary Information

Supplementary Information.
