# Supplementary material for: Circulating cell free DNA response to exhaustive exercise in average trained men with type I diabetes mellitus
Source: Sci Rep. 2021 Feb 25;11:4639. doi: 10.1038/s41598-021-84201-0 (PMC7907132; doi:10.1038/s41598-021-84201-0)
Supplement: Supplementary file 1 — Supplementary Information. [file 41598_2021_84201_MOESM1_ESM.docx]

**Circulating cell free DNA response to exhaustive exercise in average trained men with type I diabetes mellitus**

Konrad Walczak^1^, Robert Stawski^2^, Ewelina Perdas^2^, Olga Brzezinska^3^, Piotr Kosielski^4^, Szymon Galczynski^4^ ,Tomasz Budlewski^3^, Gianluca Padula^4^, Dariusz Nowak^2^*.

^1^ Department of Internal Medicine and Nephrodiabetology, Medical University of Lodz, Lodz, Poland

^2^ Department of Clinical Physiology, Medical University of Lodz, Lodz, Poland

^3^ Department of Rheumatology, Medical University of Lodz, Lodz, Poland

^4^ Academic Laboratory of Movement and Human Physical Performance, Medical University of Lodz, Lodz, Poland

* Corresponding author: E-mail: [dariusz.nowak@umed.lodz.pl](mailto:dariusz.nowak@umed.lodz.pl)

Supplemental tables

Table 1

| **cf n-DNA [ng/µl] raw data** | | | | |
| --- | --- | --- | --- | --- |
| Subject number | **T1DM volunteers** | | **Healthy controls** | |
|  | **Before exercise** | **After exercise** | **Before exercise** | **After exercise** |
|  | 13.48 | 32.65 | 4.66 | 25.04 |
|  | 9.18 | 109.45 | 3.27 | 8.16 |
|  | 2.58 | 8.66 | 1.76 | 43.86 |
|  | 3.27 | 25.79 | 6.84 | 49.35 |
|  | 4.02 | 47.92 | 2.51 | 20.37 |
|  | 2.74 | 20.98 | 1.98 | 86.44 |
|  | 5.02 | 44.44 | 3.79 | 17.57 |
|  | 3.68 | 20.98 | 1.56 | 20.37 |
|  | 5.24 | 106.27 | 3.68 | 25.79 |
|  | 2.66 | 10.96 | 4.02 | 97.27 |
|  | 4.14 | 12.33 | 2.99 | 29.01 |
|  | 5.24 | 15.16 | - | - |
|  | 3.79 | 19.20 | - | - |
|  | 3.08 | 7.47 | - | - |
| **cf mt-DNA [*10^3^ GE/mL] raw data** | | | | |
| Subject number | **T1DM volunteers** | | **Healthy controls** | |
|  | **Before exercise** | **After exercise** | **Before exercise** | **After exercise** |
|  | 50.30 | 227.41 | 786.15 | 219.94 |
|  | 137.10 | 816.94 | 571.75 | 184.87 |
|  | 11.07 | 16.16 | 293.78 | 1020.14 |
|  | 480.87 | 221.12 | 293.78 | 524.19 |
|  | 137.89 | 271.05 | 159.96 | 87.09 |
|  | 132.90 | 313.16 | 87.09 | 359.78 |
|  | 350.12 | 422.49 | 109.79 | 190.30 |
|  | 1332.34 | 359.73 | 392.43 | 359.78 |
|  | 1252.38 | 1700.25 | 539.58 | 935.28 |
|  | 729.92 | 462.86 | 126.89 | 254.19 |
|  | 374.80 | 211.53 | 539.58 | 935.28 |
|  | 55.35 | 394.15 | - | - |
|  | 183.84 | 108.61 | - | - |
|  | 422.96 | 338.07 | - | - |

Table 2

Individual results of VO2 max determination in healthy controls

| No | Anaerobic threshold | | | | | | Respiratory compensation point | | | | | | VO2max | | | | |
| --- | --- | --- | --- | --- | --- | --- | --- | --- | --- | --- | --- | --- | --- | --- | --- | --- | --- |
|  | Speed | Time | RER | HR | VO2 | %VO2max | Speed | Time | RER | HR | VO2 | %VO2max | Speed | Time | RER | HR | VO2 |
| 1 | 9.0 | 07:18 | 1.06 | 149 | 29.3 | 68.6 | 12.0 | 12:44 | 1.17 | 161 | 38.2 | 89.5 | 12.0 | 13:13 | 1.21 | 164 | 42.7 |
| 2 | 10.5 | 10:42 | 1.01 | 175 | 38.8 | 82.7 | 12.0 | 13:48 | 1.04 | 188 | 43.6 | 93.0 | 12.0 | 15:01 | 1.04 | 190 | 46.9 |
| 3 | 10.5 | 11:26 | 1.05 | 150 | 35.4 | 75.8 | 15.0 | 20:28 | 1.21 | 182 | 46.7 | 100 | 15.0 | 19:59 | 1.21 | 182 | 46.7 |
| 4 | 10.5 | 12:28 | 1.03 | 177 | 35.6 | 73.4 | 15.0 | 20:04 | 1.20 | 189 | 43.3 | 89.3 | 13.5 | 18:29 | 1.11 | 187 | 48.5 |
| 5 | 13.5 | 15:20 | 1.03 | 190 | 48.3 | 86.1 | 15.0 | 18:18 | 1.08 | 201 | 49.7 | 88.6 | 15.0 | 19:22 | 1.09 | 206 | 56.1 |
| 6 | 10.5 | 09:35 | 1.01 | 165 | 33.3 | 73.7 | 12.0 | 13:25 | 1.08 | 181 | 38.1 | 84.3 | 13.5 | 16:03 | 1.14 | 192 | 45.2 |
| 7* |  |  |  |  |  |  |  |  |  |  |  |  |  |  |  |  |  |
| 8 | 7.5 | 07:15 | 1.19 | 152 | 26.9 | 64.8 | 12.0 | 16:42 | 1.43 | 195 | 38.0 | 91.6 | 13.5 | 19:03 | 1.47 | 202 | 41.5 |
| 9 | 10.5 | 10:00 | 1.02 | 157 | 37.6 | 82.3 | 13.5 | 15:14 | 1.14 | 174 | 43.9 | 96.1 | 13.5 | 15:42 | 1.16 | 176 | 45.7 |
| 10 | 12.0 | 14:04 | 1.04 | 160 | 45.0 | 78.0 | 15.0 | 18:57 | 1.12 | 174 | 45.0 | 78.0 | 15.0 | 20:24 | 1.16 | 179 | 57.7 |
| 11* |  |  |  |  |  |  |  |  |  |  |  |  |  |  |  |  |  |

No – subject number; Speed [km/h]; Time – time from the onset of incremental test [mm:ss]; HR – heart rate [beats/min]; VO2 – oxygen consumption [ml/kgxmin]; RER – respiratory exchange ratio. * data of these two volunteers were accidentally lost from computer disk.

Table 3

Individual results of VO2 max determination in volunteers with T1DM

| No | Anaerobic threshold | | | | | | Respiratory compensation point | | | | | | VO2 max | | | | |
| --- | --- | --- | --- | --- | --- | --- | --- | --- | --- | --- | --- | --- | --- | --- | --- | --- | --- |
|  | Speed | Time | RER | HR | VO2 | %VO2max | Speed | Time | RER | HR | VO2 | %VO2max | Speed | Time | RER | HR | VO2 |
| 1 | 9.0 | 06:47 | 1.12 | 165 | 35.8 | 76.8 | 12.0 | 12:52 | 1.15 | 179 | 43.1 | 92.5 | 12.0 | 14:46 | 1.18 | 180 | 46.6 |
| 2 | 10.5 | 10:17 | 1.03 | 178 | 39.1 | 76.1 | 12.0 | 13:29 | 1.12 | 188 | 42.6 | 82.9 | 13.5 | 15:11 | 1.10 | 195 | 51.4 |
| 3 | 10.5 | 09:33 | 1.02 | 156 | 38.1 | 78.9 | 13.5 | 15:25 | 1.11 | 167 | 43.9 | 90.9 | 15.0 | 19:10 | 1.18 | 171 | 48.3 |
| 4 | 10.5 | 09:47 | 1.03 | 168 | 41.9 | 78.5 | 13.5 | 15:52 | 1.22 | 188 | 49.7 | 93.1 | 15.0 | 18:27 | 1.23 | 194 | 53.4 |
| 5 | 9.0 | 06:32 | 1.04 | 160 | 29.4 | 68.7 | 12.0 | 14:15 | 1.20 | 185 | 39.9 | 93.2 | 12.0 | 14:21 | 1.22 | 186 | 42.8 |
| 6 | 10.5 | 10:25 | 0.99 | 169 | 36.6 | 68.4 | 15.0 | 18:18 | 1.13 | 199 | 52.3 | 97.7 | 15.0 | 18:48 | 1.14 | 199 | 53.5 |
| 7 | 9.0 | 06:32 | 1.08 | 155 | 25.9 | 73.8 | 10.5 | 10:59 | 1.18 | 184 | 33.1 | 94.3 | 10.5 | 11:10 | 1.17 | 185 | 35.1 |
| 8 | 12.0 | 12:19 | 1.05 | 160 | 34.0 | 70.3 | 13.5 | 16:08 | 1.20 | 185 | 43.2 | 89.3 | 15.0 | 18:31 | 1.17 | 187 | 48.4 |
| 9 | 12.0 | 12:24 | 1.10 | 184 | 36.5 | 73.3 | 13.5 | 17:38 | 1.20 | 198 | 45.0 | 90.4 | 15.0 | 20:11 | 1.25 | 208 | 49.8 |
| 10 | 9.0 | 08:11 | 1.16 | 177 | 33.5 | 80.3 | 12.0 | 13:26 | 1.28 | 197 | 37.4 | 89,7 | 12.0 | 13:34 | 1.28 | 197 | 41.7 |
| 11 | 9.0 | 08:58 | 0.86 | 164 | 35.4 | 69.3 | 13.5 | 15:22 | 0.98 | 190 | 44.4 | 86.9 | 13.5 | 16:25 | 0.99 | 195 | 51.1 |
| 12 | 9.0 | 06:41 | 1.17 | 173 | 30.1 | 75.1 | 10.5 | 10:19 | 1.26 | 190 | 36.3 | 90.5 | 12.0 | 12:41 | 1.28 | 199 | 40.1 |
| 13 | 10.5 | 11:33 | 1.05 | 167 | 36.6 | 72.8 | 13.5 | 15:08 | 1.10 | 177 | 46.7 | 92.8 | 13.5 | 16:18 | 1.12 | 179 | 50.3 |
| 14 | 7.5 | 05:48 | 0.87 | 190 | 35.8 | 86.9 | 9.0 | 08:54 | 0.98 | 200 | 39.5 | 95.9 | 9.0 | 09:01 | 0.97 | 201 | 41.2 |

No – subject number; Speed [km/h]; Time – time from the onset of incremental test [mm:ss]; HR – heart rate [beats/min]; VO2 – oxygen consumption [ml/kgxmin]; RER – respiratory exchange ratio

Table 4

Effect of glucose concentration on neutrophil extracellular traps (NETs) formation in suspensions of neutrophils isolated from healthy subjects – review of previous in vitro studies

| Experimental conditions | Stimulation of NETs formation by glucose concentration | Ref |
| --- | --- | --- |
| Incubation with glucose concentrations of 5.5 , 10, 20 and 30 mmol/L for 2 h at 37°C | >10 mmol/L | 22 |
| Incubation with glucose concentrations of 5.5 , 10, 20 and 30 mmol /L for 24 h at 37°C | 20 and 30 mmol/L | 18 |
| Incubation with glucose concentrations of 5.5 , 15 , 25 and 35 mmol/L for 2 h at 37°with 5% CO_2_ | 25 and 35 mmol/L | 29 |
| Incubation with glucose concentrations of 5 and 25 mmol/L for 24 h at 37°C | 25 mmol/L | 19 |
| Incubation with glucose concentrations of 5.5 , 10 , 15 , 20 , 25 and 30 mmol/L for 24 h at 37°C. | 15 and 20 mmol/L | 30 |

Table 5

Median percentage increments in circulating cell free nuclear DNA and selected metabolic and muscle damage markers in response to exhaustive treadmill run in T1DM subjects and healthy controls

| Variable | Bout of exhaustive treadmill run | |
| --- | --- | --- |
|  | T1DM subjects | Healthy controls |
| cf n-DNA | 596±504* (438) | 1266±1242† (712) |
| Lactate | 381±271* (324) | 615±643 (351) |
| CK | 22 ± 15‡ (19) | 30±28‡ (30) |
| Creatinine | 20±10‡ (17) | 39±19**‡ (36) |
| Urea | 7±4 (6) | 10±6 (10) |

cf n-DNA – cell free nuclear DNA, CK- creatine kinase. Results are expressed as mean and standard deviation (and median in parentheses). Other details as for Table 2. * vs corresponding values obtained for CK , urea and creatinine, p< 0,05. † vs all corresponding values, p<0.05 . ‡ vs corresponding value of urea, p< 0.05, ** vs corresponding value in T1DM subjects, p<0.05.

Supplemental figures

Fig. 1. Correlation plot between Δ cf n-DNA and Δ CK in healthy controls. Δ – exercise-induced increment, cf n-DNA – circulating cell free nuclear DNA, CK – creatine kinase.

Fig. 2. Correlation plot between Δ cf n-DNA and pre-exercise creatinine in healthy controls. Δ – exercise-induced increment, cf n-DNA – circulating cell free nuclear DNA.

Fig. 3. Correlation plot between Δ cf n-DNA and pre-exercise WBC in healthy controls. Δ – exercise- induced increment, cf n-DNA – circulating cell free nuclear DNA, WBC white blood cells.

Fig. 4. Correlation plot between Δ cf n-DNA and post-exercise WBC in healthy controls. Δ – exercise- induced increment, cf n-DNA – circulating cell free nuclear DNA, WBC – white blood cells.

Fig. 5. Correlation plot between Δ cf n-DNA and Δ WBC in healthy controls. Δ – exercise-induced increment, cf n-DNA – circulating cell free nuclear DNA, WBC white blood cell count.

Fig. 6. Correlation plot between Δ cf n-DNA and pre-exercise neutrophils in healthy controls. Δ – exercise-induced increment, cf n-DNA – circulating cell free nuclear DNA.

Fig. 7. Correlation plot between Δ cf n-DNA and post-exercise neutrophils in healthy controls. Δ – exercise-induced increment, cf n-DNA – circulating cell free nuclear DNA.

Fig. 8. Correlation plot between Δ cf n-DNA and Δ neutrophils in healthy controls. Δ – exercise- induced increment, cf n-DNA – circulating cell free nuclear DNA.

Fig. 9. Correlation plot between Δ cf n-DNA and pre-exercise neutrophils in T1DM subjects. Δ – exercise-induced increment, cf n-DNA – circulating cell free nuclear DNA.

Fig. 10. Correlation plot between Δ cf n-DNA and post-exercise neutrophils in T1DM subjects. Δ – exercise-induced increment, cf n-DNA – circulating cell free nuclear DNA.
